# An evaluation of the Just Five program, a flexible digital approach to adult substance use education

**DOI:** 10.1371/journal.pone.0277112

**Published:** 2022-11-10

**Authors:** Leslie Damesek Litsky, Stephen D’Antonio, Erika Bonnevie

**Affiliations:** 1 Shatterproof, Norwalk, Connecticut, United States of America; 2 Independent Researcher, San Diego, California, United States of America; University Kebangsaan Malaysia, MALAYSIA

## Abstract

Substance use is a steadily worsening crisis, yet there is limited evidence on the effectiveness of brief educational programs related to substance use disorders (SUD). To address this, the Just Five digital program launched in 2019 with six educational lessons about SUD, each five minutes long. Just Five is delivered through various organizations, including employers as part of workplace wellness, health plans that offer it to members, and to community-based organizations and populations they serve. This study evaluated the effectiveness of the program across 10 organizations in the initial launch. A cross-sectional survey was given to Just Five viewers to establish changes in perceived knowledge and attitudes toward SUD. Results were analyzed among the overall sample; a sub-analysis compared results within the three types of organizations taking part. Between December 2019 and September 2021, 2,749 baseline and 397 follow-up surveys were collected. Significant improvements were observed across almost all questions asked, with the largest improvements in respondent confidence to recognize signs of addiction (52.5% baseline; 87.7% follow-up); knowledge about ways to reduce the risk of addiction (54.1% baseline; 86.5% follow-up); and knowledge of how to help someone with addiction (38.7% baseline; 81.7% follow-up). All trends were replicated in sub-analyses. Substance use has severe societal impacts, and there is a need for effective programs that can be quickly implemented across various environments at large scale. Just Five is a potentially effective tool to increase perceived knowledge and improve attitudes toward SUD.

## Introduction

In the United States, substance use is an ongoing crisis that is steadily worsening, particularly since the start of the COVID-19 pandemic. At present, drug overdoses are the top cause of accidental death [1]. There were over 100,000 fatal overdoses in the U.S. from April 2020 to April 2021, the highest number of overdose deaths ever recorded in a 12-month period [2]. Experts expect the substance use crisis to continue, as the stress and isolation brought on by the pandemic converges with the rise of illicit fentanyl [3]. Substance use disorders include addiction to drugs and alcohol [4]. A substance use disorder (SUD) is a mental disorder that affects a person’s brain and behavior, leading to a person’s inability to control their use of substances such as legal or illegal drugs, alcohol, or medications. Symptoms can range from moderate to severe, with addiction being the most severe form of SUDs. The risk factors for SUD have been well studied, with various factors contributing to the likelihood of experiencing addiction, including age, genetics, environmental stressors, childhood trauma, and gender [5–7]. SUD can have significant consequences that impact both the individual and community, including higher healthcare costs, the spread of infectious disease, drug-related crime, interpersonal violence, unintended pregnancy, and family stress [8]. Substance use also impacts the workplace, negatively affecting businesses through billions of dollars lost each year due to lost productivity, workplace accidents and injuries, employee absenteeism, low morale, and increased illness [9].

Despite the clear impact of SUD on society, there is limited evidence on the effectiveness of brief adult educational interventions. However, a review of the available literature suggests brief interventions can have an impact on a person’s actions and attitudes around substance use, particularly related to alcohol consumption [10]. Many programs have been focused on youth prevention, with brief educational interventions around substance use showing positive impacts among adolescents [11]. A larger body of literature focused on adults has shown promise in the use of brief interventions for substance use treatment, and to increase knowledge of SUD among counselors and clinicians [12–14]. Research has also shown success in embedding adult education programs into workplaces, with limited evidence to suggest promise in promoting drug education specifically [15–18].

The CDC provides guidance on effective Workplace Health Models, suggesting that the workplace is one important setting for health protection, health promotion, and disease prevention programs, but does not mention SUD [19]. There is limited information on best practices for general adult SUD education programs that can be implemented across various contexts, including workplaces and within communities. This is particularly true for programs that can be digitally implemented. Digital educational interventions have the benefit of being scalable to various contexts, can be taken privately, and can be delivered when in-person activities are limited (for example, during a global pandemic).

In response to this gap in evidence, the Just Five digital SUD education program was created and launched in 2019 by Shatterproof, a national nonprofit organization dedicated to reversing the addiction crisis in the U.S. Just Five is designed for companies interested in educating their employees about substance use disorders. The program, offered in English and Spanish, includes six lessons, all five minutes or less in length, designed to educate viewers with clinically accurate information about addiction and how to support their own or a loved one’s health. Each organization or company receives a personalized landing page with both the Just Five educational program, as well as other employer or community resources. To encourage participation, the program is delivered through an open website, without a requirement to enroll or enter any identifying information to access the lessons. Lessons 1–3 focus on general addiction education, including the science of addiction, risk factors for addiction, and specific information on opioids. Lessons 4–6 provide actionable steps for those impacted to be able to identify signs of addiction, access evidence-based treatment, and support loved ones through to recovery. All lessons are cited and include additional resources for those interested in reading more about a topic. The program is sponsored and promoted by various types of organizations, including: 1) employers who provide Just Five to their employees through employer wellness programs, 2) health plans who provide Just Five to their subscribers, or 3) through community-based organizations who offer Just Five to the general community and members they serve. Organizations are provided with a Just Five toolkit that offers suggestions on how to integrate the program into other health educational initiatives. More information about the Just Five program can be found on the website justfive.org. More information about the lessons can be found in S1 File. This study will evaluate the potential effectiveness of the Just Five program, distributed across 10 organizations that took part in the initial launch of the program.

## Methods

To establish knowledge and attitudes toward SUD before engaging in Just Five, two online surveys were offered to each individual participating in the program, one immediately. before the first lesson, and one immediately after the final lesson. Eligibility criteria for the survey included being over 18 years old, receiving an invitation to take part in the Just Five program from either an employer, health plan, or community organization, and being English-speaking (given that all 10 organizations participated in the English-language version of the program only). Personally identifiable information, including demographic information, was not collected from respondents, and all respondents were identified with a unique identifier for analysis. Before participating in the surveys, participants were provided with an informed consent statement, informing them that their responses were voluntary and confidential. Respondents were not required to answer questions and could quit the survey at any time. Given that the survey presented no more than minimal risk of harm to participants and no identifiable information was collected, the need for documented written consent was waived by the Institutional Review Board Advarra, which deemed this study to be exempt from review. Their submission of survey answers was considered consent. Immediately after completion of the final module, individuals were requested to complete the follow-up survey. The surveys consisted of questions adapted from existing research on substance use and stigma [20–23], and covered themes related specifically to the content delivered within each module, including: 1) Knowledge about substance use, susceptibility to addiction, and treatment options; 2) Attitudes toward recovery and perceptions of morality; 3) Recognizing the signs of addiction and 4) Awareness of how to help a loved one. The baseline and follow-up surveys contained the same questions, to measure change over time.

Due to privacy restrictions from the organizations taking part in the Just Five program, it was not possible to link responses from the baseline and follow-up datasets. Therefore, the sample likely contained both longitudinal and cross-sectional respondents. Incentives for survey participation varied, depending on the policy of the organization. One community-based organization provided $5 coffee cards for survey participation, while all others did not provide any incentive, per organization policies. Given that the Just Five program enrolls organizations and participants on a rolling basis, surveys were delivered at differing times for each organization, depending on the organization’s enrollment into the program, or new staff or plan members start date (in the case of employers and health plans). Analysis was conducted on baseline surveys across 10 organizations. Demographic information was not available for all respondents taking part in the program and was therefore not included in the analysis. Data were analyzed both among the overall sample of survey responses and were also segmented according to the three types of organizations that took part in the program. Data were segmented by employers who offered Just Five to their employees through a wellness program (four sites, termed ‘Employer’ throughout the analysis), health plans who made the program available to health plan subscribers (two sites, termed ‘Health Plan’ throughout), and community-based organizations who offered the program to the general communities they serve (four sites, termed ‘CBO’ throughout).

Questions were all asked on a five-point Likert scale, from “strongly agree,” “agree,” “neither agree nor disagree,” “disagree” and “strongly disagree.” For the purposes of analysis, “strongly agree” and “agree” were combined into one category (Agree), and “strongly disagree” and “disagree” were combined into one category (Disagree). Negative questions were asked to prevent potential response set bias. For negative questions, disagreement is presented in the tables below, and noted where relevant. For analysis, organizations were segmented into three categories, based on the type of individual they reached. Data were compared using a two-sided Pearson Chi-square test using IBM SPSS Statistics software and significance was assessed at a p-value of ≤.05.

## Results

A total of 2,749 baseline surveys were collected between 12/15/2019 and 9/29/2021 and 397 follow-up surveys were collected between 2/4/2020 and 9/28/2021. Health Plan respondents comprised 42.8% of all survey respondents (1,177 baseline; 171 follow-up), followed by Employer respondents with 32.0% of surveys (909 baseline; 97 follow-up), and CBO respondents with 25.2% of surveys (663 baseline; 129 follow-up). Respondents showed significant improvements from baseline to follow-up, across almost all questions asked (Table 1). The largest improvements were observed in respondent confidence to recognize the signs of drug or alcohol addiction (52.5% baseline; 87.7% follow-up); knowledge about ways to reduce the risk of addiction (54.1% baseline; 86.5% follow-up); and knowledge of how to help someone with drug and alcohol addiction (38.7% baseline; 81.7% follow-up). Questions with the highest agreement at baseline and follow-up included agreement that virtually anyone can become addicted to drugs and alcohol (89.7% baseline; 94.9% follow-up), and disagreement that there are no effective treatments for addiction (89.5% baseline; 93.4% follow-up). Morality-based attitudes around the lack of moral strength (65.1% baseline; 79.7% follow-up) and fault for addiction (60.7% baseline; 79.3% follow-up) showed less improvement, though they were still statistically significant.

**Table 1:** Agreement with survey questions among overall respondents participating in the Just Five program, from baseline to follow-up.

	Baseline		Follow-up		
Question*	n	%	n	%	p-value
I feel confident that I could recognize the signs of drug or alcohol addiction.	1466	53.5%	342	87.7%	≤.001
I know ways to reduce the risk of addiction.	1472	54.1%	315	86.5%	≤.001
I know how to help if I suspect someone is addicted to drugs or alcohol.	1054	38.7%	308	81.7%	≤.001
A lack of moral strength plays a large part in drug and alcohol addiction.^+^ *(Disagree)*	1776	65.1%	294	79.7%	≤.001
There are no effective treatments to help people addicted to drugs and alcohol.^+^ *(Disagree)*	2442	89.5%	342	93.4%	.001
People who are addicted to drugs or alcohol are at fault for their addiction.^+^ *(Disagree)*	1660	60.7%	315	79.3%	≤.001
It is ok for teenagers to try drugs and alcohol, because it’s a part of growing up. ^+^ *(Disagree)*	2201	80.7%	354	89.2%	≤.001
People who are addicted to drugs and alcohol can fully recover.	1913	70.1%	311	78.7%	.001
Virtually anyone can become addicted to drugs or alcohol.	2446	89.7%	375	94.9%	.004
Addiction is a chronic medical illness like diabetes, arthritis, or heart disease.	2248	81.9%	368	93.4%	≤.001

*Unless noted, all questions show respondents who reported “strongly agree” or “agree”

^+^Questions were originally asked in the negative; this percentage shows disagreement.

Respondents were then categorized by the type of organization that took part in the Just Five program (CBO, Employer, Health Plan) to examine whether improvements in questions among the overall sample held true for each type of organization engaged in the program (Table 2). Overall trends shown above were reflected in this sub-analysis. All sites showed significant improvements in feeling confident to recognize the signs of drug or alcohol addiction, with between 50–60% agreement at baseline and 80–90% at follow-up, across all three categories (p≤.001 for all three). Similar significant improvements were observed in knowing how to reduce the risk of addiction, with 50–55% agreement at baseline to 80–90% agreement at follow-up (p≤.001 for all three). Knowledge of how to help someone with addiction showed further significant increases, from 35–40% agreement at baseline to 80–83% agreement at follow-up (p≤.001 for all three). Agreement that addiction is a chronic medical illness also showed significant improvements across all three groups, from about 80–83% at baseline to 93–95% at follow-up.

**Table 2:** Agreement with survey questions, from baseline to follow-up by category of organization participating in the Just Five program.

	Baseline		Follow-up		
	n	%	n	%	p-value
*I feel confident that I could recognize the signs of drug or alcohol addiction*.					
CBO	416	60.3%	135	89.1%	≤.001
Health Plan	586	50.0%	148	89.2%	≤.001
Employees	464	52.9%	59	83.3%	≤.001
*I know ways to reduce the risk of addiction*.					
CBO	353	51.3%	130	91.1%	≤.001
Health Plan	644	55.5%	131	86.2%	≤.001
Employer	475	54.5%	54	80.9%	≤.001
*I know how to help if I suspect someone is addicted to drugs or alcohol*.					
CBO	274	39.5%	121	83.1%	≤.001
Health Plan	419	36.0%	129	81.1%	≤.001
Employer	361	41.6%	58	80.9%	≤.001
*A lack of moral strength plays a large part in drug and alcohol addiction*.^+^ *(Disagree)*					
CBO	457	65.3%	107	74.2%	.150
Health Plan	773	66.4%	131	84.5%	≤.001
Employer	546	63.4%	56	78.9%	.004
*There are no effective treatments to help people addicted to drugs and alcohol*.^+^ *(Disagree)*					
CBO	619	88.8%	134	92.7%	.027
Health Plan	1039	89.1%	145	95.4%	.021
Employer	784	90.6%	63	91.3%	.594
*People who are addicted to drugs or alcohol are at fault for their addiction*.^+^ *(Disagree)*					
CBO	433	62.4%	112	72.9%	.076
Health Plan	700	60.0%	139	81.3%	≤.001
Employer	527	60.5%	64	84.5%	≤.001
*It is ok for teenagers to try drugs and alcohol*, *because it’s a part of growing up*.^+^ *(Disagree)*					
CBO	517	73.8%	129	83.7%	.054
Health Plan	944	81.0%	160	93.6%	≤.001
Employer	740	85.3%	65	88.7%	.282
*People who are addicted to drugs and alcohol can fully recover*.					
CBO	515	74.3%	125	81.3%	.212
Health Plan	809	69.2%	126	74.1%	.042
Employer	589	68.2%	60	83.5%	.005
*Virtually anyone can become addicted to drugs or alcohol*.					
CBO	625	89.4%	145	96.9%	.027
Health Plan	1040	89.3%	160	94.1%	.145
Employer	781	90.2%	70	93.8%	.376
*Addiction is a chronic medical illness like diabetes*, *arthritis*, *or heart disease*.					
CBO	586	83.4%	140	93.0%	.006
Health Plan	961	81.9%	157	92.9%	.001
Employer	701	80.9%	71	94.8%	.003

^+^Questions were originally asked in the negative; this percentage shows disagreement.

Across several questions, results showed significant improvements among Health Plan and Employer groups, and non-significant improvements among CBOs. This included disagreement that a lack of moral strength plays a part in addiction, with around 65% at baseline and more than 80% at follow-up among Health Plan and Employer respondents. The belief that people can fully recover showed 69% agreement at baseline, with 74% at follow-up for Health Plan and 84% for Employer respondents. Finally, these two groups showed significant improvements in beliefs that people are not at fault for their addiction, with 60% at baseline to more than 80% at follow-up.

Other questions showed varying trends. CBO and Health Plan respondents showed significant improvements on beliefs about the effectiveness of treatment (89% at baseline to 93–95% at follow-up), Health Plan respondents showed significant improvements in believing that it is not ok for teenagers to try drugs and alcohol (81% baseline; 94% follow-up), and CBO respondents showed significant improvements on beliefs that virtually anyone can become addicted (89% baseline; 97% follow-up).

## Discussion

Awareness and education around substance use disorders (SUD) is critical to improve the health and wellbeing of individuals and communities. This evaluation of the Just Five program suggests that the program may be a useful tool to increase perceived knowledge and improve attitudes toward SUD across various contexts—as part of an employer wellness program, when sponsored by a health plan to its subscribers, and when promoted by community-based organizations. Results from three questions were particularly notable: the largest increases were shown in feeling confident around recognizing the signs of addiction (which increased 34.2 percentage points in the overall sample), knowing ways to reduce the risk of addiction (which increased 32.4 percentage points) and knowing how to help if an acquaintance is living with addiction (which increased 43.0 percentage points). These dramatic improvements suggest that respondents may be more able to support loved ones after taking the Just Five program [24]. Although this pilot study carries limitations, especially due to the sample size at follow-up and the inability to match longitudinal responses, these substantial increases over time are still important to note, particularly given that trends held firm within the sub-analysis by the three types of groups. Additional studies are needed to examine whether future applications of the Just Five program can sustain and replicate these high levels of improvement across different contexts.

Although substance use severely impacts individuals, organizations, and society, there is a gap in evidence on programs that can be effectively delivered across various environments. Within the workplace, Just Five can fill a critical gap in SUD education as a part of employer wellness programs. In the past, employer wellness programs have focused on promoting health screenings, chronic disease prevention and management, flu shots, and mental health and stress management [25]. These programs are frequently justified by employer cost savings, resulting from a healthier workforce [26]. The Society for Human Resources Management (SHRM) recently highlighted SUDs as “the last taboo topic in corporate America” [27]. SAMHSA’s directive for developing a drug-free workplace program focuses on the process of developing a program with resources related to accessing drug-testing information and laboratories but does not provide example programs [28]. There is a need for evidence-based education programs that can be easily adapted and implemented across work places.

In the past several decades, health plans have placed an increasing emphasis on wellness programs as a way of supporting the health of their member base and reducing costs [29]. To date, healthcare-related substance misuse programs have predominantly focused on the clinical space, through inpatient and outpatient SUD assessments, provider training, and continuing education for healthcare professionals. There is less information on programs that health plans can promote to their member base. Yet for every dollar spent on wellness programs, medical costs fall by more than $3.00, making investments in SUD education an important strategy for health plans to reduce their costs [26]. This is particularly important given inflation and rising health spending both at the federal and individual level. While individual health spending slowed throughout the pandemic due to decreases in service utilization at the start of the pandemic, spending is likely to substantially increase as hospitals return to their normal state [30]. Effective adult substance use education is more important than ever for health systems, given the severe morbidity and mortality and high healthcare costs associated with SUD [31].

Finally, the flexibility and digital nature of Just Five also allows for its delivery to the public through community organizations. There are few brief, evidence-based substance use programs are available to reach adults in the community. Community substance use education programs are usually geared toward school-aged students through longer-term curricula focused on prevention [32, 33]. Or, they are delivered with the intention of reaching people who are living with addiction and encouraging them to seek treatment or to educate parents on how to prevent addiction among their children [34–36]. Future research should examine the potential impact of Just Five when delivered through various types of communities to understand differences in impact by demographic group, including impact among Spanish speakers, or geographic area served by the CBO.

## Limitations

This study carries with it some limitations. Due to restrictions from the organizations taking part in the Just Five program, it was not possible to determine differences between longitudinal and cross-sectional respondents. Therefore, is it not possible to definitively associate changes to participation in the program. It is possible that survey respondents who took the follow-up survey already had better views toward addiction, or that survey respondents answered the surveys according to a response bias. For most organizations that participated in the program, there was a large drop-off from baseline to follow-up, likely due to the fact that most organizations taking part in the program could not incentivize the surveys. Particularly when analyzed by the type of organization involved in Just Five, this low response rate may have limited the ability to find significant improvements over time. Future researchers should undertake a longitudinal study to link changes more definitively to the program. Despite these limitations, we feel this study fills important gaps in evidence on brief interventions to address addiction.

## Conclusion

The Just Five program shows great potential as an important brief adult awareness program on substance use. The evaluation of 10 sites enrolled in the Just Five program showed significant increases in perceived knowledge, attitudes and levels of confidence in recognizing and helping with substance use issues. This suggests that the program may be an important addition to the current evidence base around tools for adult substance use education. As the United States now shifts attention away from the pandemic, it is important to have as many tools available as possible to help fight the growing substance use crisis.

## Supporting information

S1 File Just five lessons and sample images. (DOCX)
