# Supplementary material for: An evaluation of the Just Five program, a flexible digital approach to adult substance use education
Source: PLoS One. 2022 Nov 10;17(11):e0277112. doi: 10.1371/journal.pone.0277112 (PMC9648762; doi:10.1371/journal.pone.0277112)
Supplement: S1 File — (DOCX) [file pone.0277112.s001.docx]

## S1. Supplemental File: Just Five Lessons and Sample Images

| **Topic** | **Description** | **Sample Imagery** |
| --- | --- | --- |
| 1. The Science of Addiction | *Addiction is not a choice or a character flaw. It is a chronic medical condition that changes the brain. In this lesson, learn how a substance use disorder impacts the body and the brain.* | **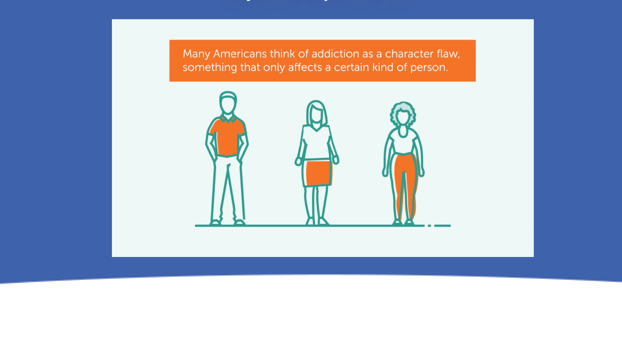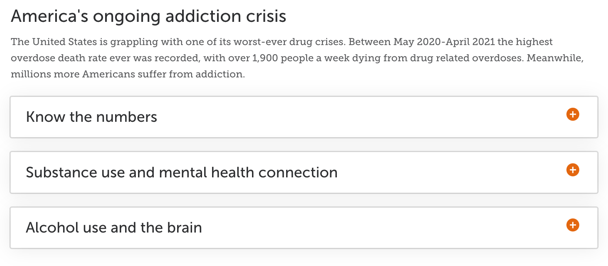** |
| 2. Risks/ Susceptibility to Addiction | *In this lesson, you will learn who is at risk for addiction and how it varies from person to person based on age of first use, genetics, and environment.* | **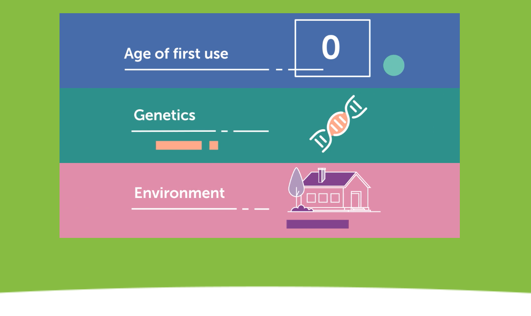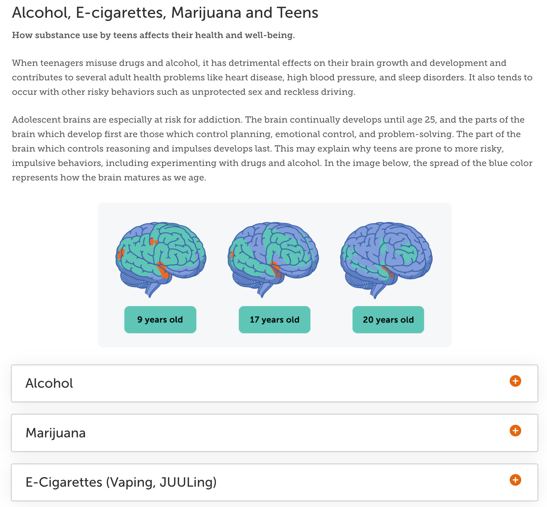** |
| 3. Opioids | *Opioids are often prescribed for treating certain kinds of pain, but they can carry serious risks. In this lesson, learn about common opioids, find alternatives for pain management, and discover questions you should ask your doctor before taking prescription painkillers.* | **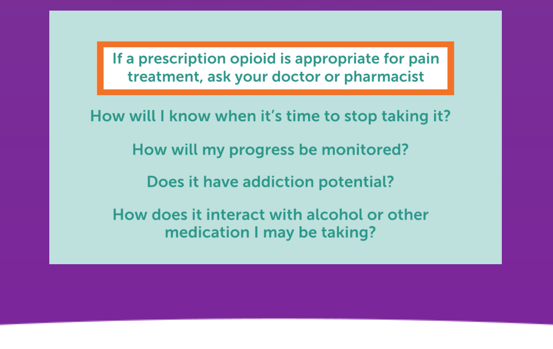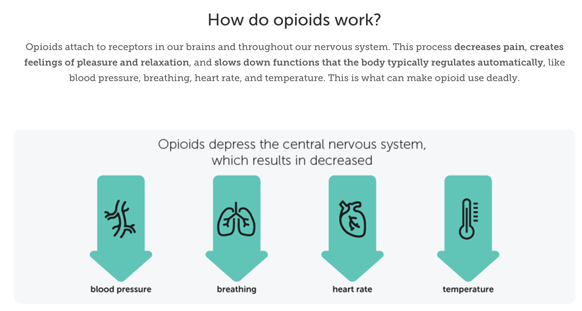** |
| 4.  Signs of Addiction and Treatment Options | *In this lesson you will learn how to spot the signs and symptoms of a substance use disorder. You can also take a simple, one-minute self-assessment to determine if you or a loved one has a substance use disorder.* | **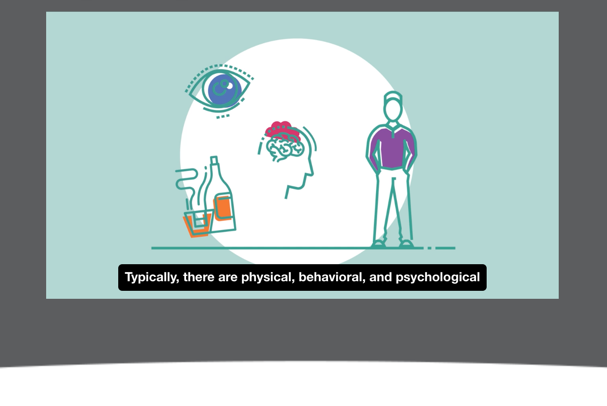**  **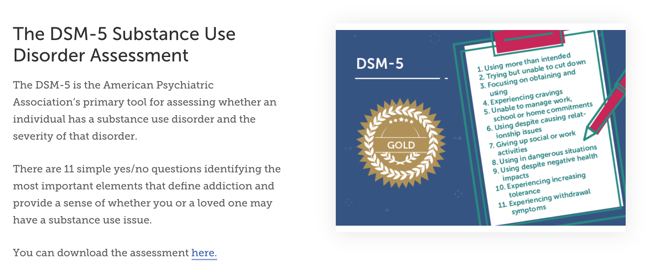** |
| 5. Helping a Loved One with Addiction | *Coping with another’s substance use can take a devastating mental toll. In this lesson, learn how to help someone with addiction and how to support a friend or coworker managing addiction in their family.* | **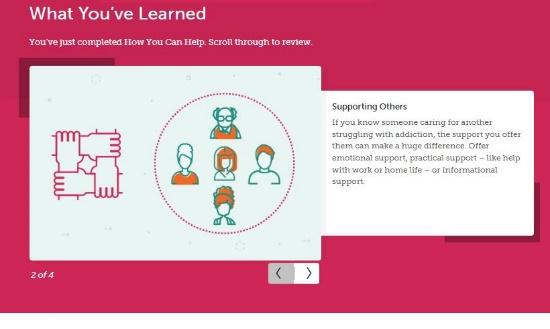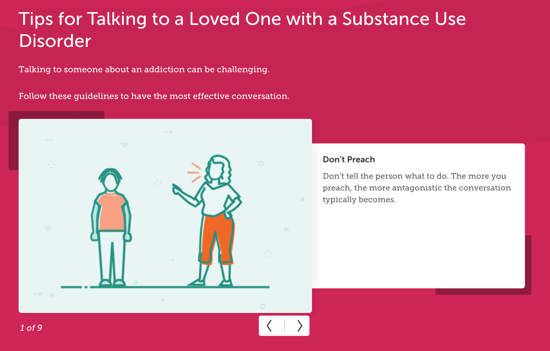** |
| 6. Recovery | *Recovery from addiction is a journey that transforms one’s life from deep despair to great possibilities. In this lesson, learn how recovery means regaining control over one’s life and see how it can be enormously gratifying.* | **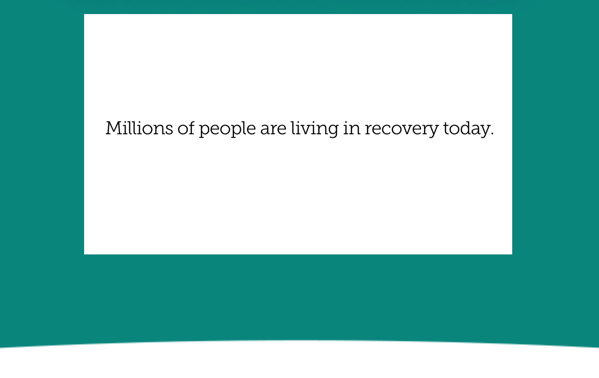**  **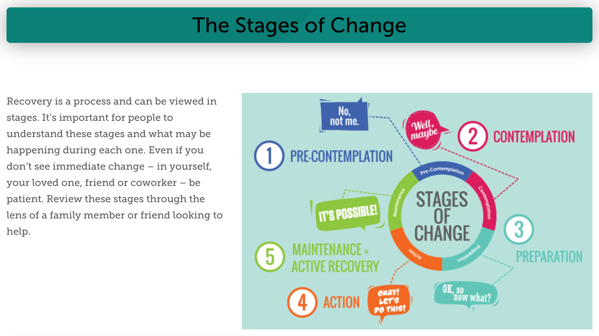** |
